# Land Use Diversification to Enhance Livelihood Resilience in the Face of Climate Change in Ethiopia

**DOI:** 10.1155/tswj/8444736

**Published:** 2026-09-15

**Authors:** Shibire Bekele Eshetu, Meselu Tegenie Mellaku, Haftom Hagos, Katharina Löhr, Negasi Solomon, Stefan Sieber

**Affiliations:** ^1^ Leibniz Centre for Agricultural Landscape Research (ZALF), Müncheberg, Germany; ^2^ Thaer-Institute for Agricultural and Horticultural Sciences Agricultural Economics, Humboldt Universität zu Berlin, Berlin, Germany, hu-berlin.de; ^3^ Wondo Genet College of Forestry and Natural Resources, Hawassa University, Hawassa, Ethiopia, hu.edu.et; ^4^ Faculty of Environmental Sciences and Natural Resource Management (MINA), Norwegian University of Life Sciences (NMBU), Ås, Norway, nmbu.no; ^5^ Inst. for Agricultural Engineering and Bioenergy, University of Kassel, Kassel, Germany, uni-kassel.de; ^6^ Institute of Climate and Society (ICS), Mekelle University, Mekelle, Ethiopia, mu.edu.et; ^7^ University of Sustainable Development, Eberswalde, Germany; ^8^ Tigray Institute of Policy Studies, Mekelle, Tigray, Ethiopia; ^9^ Department of Land Resource Management and Environmental Protection, College of Dryland Agriculture and Natural Resources, Mekelle University, Mekelle, Ethiopia, mu.edu.et

**Keywords:** agroforestry, diversification, food security, land use, woodlots

## Abstract

This study examines how different land use systems are associated with resilience to drought‐induced food insecurity in Ethiopia using a comparative case study of three districts representing monocropping (Bale), agroforestry (Sidama), and woodlot‐based systems (Awi). The analysis integrates crop damage data from the Central Statistical Agency (CSA) with food aid priority classifications and postdrought food security outcomes from the Integrated Food Security Phase Classification (IPC) and food aid priority area map from World Food Program (WFP), Inter‐Agency Humanitarian Evaluation (IAHE) and United Nations Office for the Coordination of Humanitarian Affairs (OCHA). Results show marked differences in drought impacts across systems. During the 2015–2016 drought, Bale (monocropping) experienced the highest crop losses (54,354 ha; 0.29 ha per affected person), followed by Sidama (agroforestry) (6629 ha; 0.19 ha per person) and Awi (woodlot) (3802 ha; 0.02 ha per person). Normalized against projected 2016 zone populations, Bale also records the highest area loss per 1000 inhabitants (30.8 ha, vs. 3.1 in Awi and 1.8 in Sidama), although the share of the zone population reporting crop damage was highest in Awi (15.4%). Postdrought IPC classifications further indicate higher food insecurity in Bale, where 60% of the population was classified in Phase 2 or above, compared with 30% in Sidama, whereas Awi did not meet the threshold for IPC classification. These patterns suggest that, within the studied contexts, diversified land use systems particularly agroforestry‐ and woodlot‐based practices are associated with lower exposure to drought impacts and reduced food insecurity. However, these relationships are context‐specific and reflect the combined influence of land use systems and broader agroecological and socioeconomic conditions. In particular, mean annual rainfall differs more than twofold across the study districts, and the woodlot district is also the wettest, so baseline moisture availability cannot be separated from land use configuration as an explanation of the observed differences. The findings are therefore indicative rather than causal, but they highlight the potential role of land use diversification as part of strategies to enhance resilience to climate variability.

## 1. Introduction

Future food production is highly vulnerable to climate change, posing a serious threat to global and regional food security [1, 2]. Climate variability and extreme events directly impact food production, with global mean temperature rises of each degree Celsius reducing crop yields by 3.1%–7.45% of the global yield [3]. This occurs through both direct effects such as heightened water stress and increased incidence of pests and diseases and indirect effects on agricultural productivity [4]. Sub‐Saharan African countries are among the most affected by climate change due to lower socioeconomic status, which limits their adaptive capacity to rapidly growing climate change effects [5]. Furthermore, the region′s heavy reliance on rain‐fed agriculture, which employs between 60% and 90% of the active labor force, exacerbates this vulnerability [6, 7].

Ethiopia′s agricultural sector dominates its economy, accounting for about 32.7% of GDP and employing roughly 85% of the population [8]. Despite rapid growth since 2000, agricultural productivity remains low, challenged by unpredictable rainfall patterns, rising temperatures, and land degradation [9, 10]. Land degradation, characterized by soil fertility loss, erosion, and natural resource depletion contributes significantly to declining productivity [11]. Moreover, urbanization, demographic changes, and governance challenges further affect household food security [12–14]. These climatic and socioeconomic stressors severely threaten the livelihoods of smallholder farmers dependent on natural resources and rain‐fed agriculture [15]. Furthermore, urbanization [13], socioeconomic, and demographic aspects [14, 16], and policy and governance [12] influence the food security of households.

Climate change and its variability impacts are severe for the people who depend on natural resources and rain‐fed agriculture [15]. As a result of the strong El Niño that occurred from late 2015 to early 2016, a precipitation shift has been recorded in the Sahel region [17]. This had impacted Ethiopia′s main rainy season locally called “kiremt,” to be late and below normal conditions marking one of the driest years in large parts of the country. Consequently, widespread significant crop failure occurred in the country and according to the US Department of State briefing on March 2016 [18], the government of Ethiopia called for emergency assistance for about 10 million people. Suryabhagavan [19] confirmed that the most severe droughts Ethiopia experienced are linked to the failure of rainfall from June to September which constitutes 65%–95% of the total rainfall.

As a solution, farmers increasingly adopt land diversification as a crucial adaptation strategy to mitigate the adverse effects of climate change on agricultural productivity and livelihood resilience. Land use diversification is a critical strategy to enhance resilience and mitigate the adverse impacts of climate change, especially in vulnerable regions such as Ethiopia. By integrating multiple land uses such as crop diversification, agroforestry, and woodlot establishment, this approach helps to spread risks, improve soil health, and increase ecosystem services including carbon sequestration [20, 21]. Diversification reduces dependency on monoculture systems that are highly susceptible to climate variability and extreme weather events, thereby stabilizing food production and improving livelihood security for smallholder farmers [22]. Furthermore, diversified land use systems contribute to climate change adaptation by enhancing biodiversity, improving water retention, and restoring degraded lands, which in turn bolster the capacity of farming households to withstand climatic shocks [23, 24].

Increase of off‐farm work, planting drought‐tolerant crop varieties, early plantings, and intercropping were also used as key ex‐ante adaptation strategies toward climate variability‐induced risks like drought, floods, and crop pests and diseases [25]. Furthermore, rural farm households are responding to climate variability‐induced risks in various ways, which include conversion of agricultural land use to woodlot [26, 27] and adoption of agroforestry systems [28]. Small‐scale plantations practiced by farm households meet household for wood products demand and income, accounting for the major share of plantation forestry in Ethiopia [29]. In addition to the role of woodlot management in improving livelihood performance, the contribution to meeting the 30% forest cover target by 2030 is crucial. Studies also indicate that agroforestry can help farmers build resilience by contributing toward enhancing the capacity of the households to deal with various shocks [30–32]. Agroforestry, as a diversified land use system potentially helps in improving soil health productivity, livelihood and food security [21].

In most parts of African countries, crop and land use diversification has been known as one of the adaptation strategies whereby tree planting and agroforestry systems are known to play a great role in climate change adaptation strategies [21]. Agroforestry techniques can be perfected to cope with the new conditions that are anticipated under drier conditions and higher population density; they lead to an increase in the amount of organic matter in the soil thereby improving agricultural productivity and reducing the pressure on forests [21, 33].

Different farming practices and land use diversification strategies respond unequally to climate variability and climate‐induced shocks. Understanding the comparative advantages of these practices is essential for guiding land use planners and farmers toward options that enhance resilience to climate risks. This study, therefore examines how diversified land use systems specifically monocropping, agroforestry, and woodlots contribute to farm households′ livelihood resilience during climate shocks, using the 2015–2016 drought as a reference event. The study is aimed at (i) analyzing land use and land‐cover patterns in the selected case‐study sites during the drought period; (ii) assessing the vulnerability of smallholder farmers to climate‐induced risks; and (iii) evaluating the relationship between land use diversification and livelihood resilience, measured in terms of reduced reliance on food‐aid support during the 2015–2016 drought.

## 2. Conceptual Framework

Ethiopia′s agricultural sector is highly susceptible to the impacts of climate change, primarily due to its dependence on rainfall, agroecology, and lower adaptive capacity [34, 35]. The 2015–2016 Ethiopian drought, exacerbated by a strong El Niño event, highlighted the severe impacts of climate change on the country′s predominantly rain‐fed agricultural sector. The drought has severely affected the agricultural productivity of the country [36, 37]. Rainfall deficit was the leading cause of crop loss, livestock mortality, and increased dependency on humanitarian aid [37]. The drought highlighted the vulnerability of rain‐fed agriculture‐reliant households to the impacts of climate change, emphasizing the urgent need for adaptive strategies to mitigate future risks and safeguard livelihoods in the face of changing climatic conditions.

Studies suggest that climate change adaptation to reduce the impact of climate change on food security is of high priority for smallholder farmers in Ethiopia. Hereby adaptation can relate to/aim for (i) developing capacity to cope with the inevitable damage, (ii) reducing the exposure to the risk of damage, and (iii) taking advantage of new opportunities. A review of Africa′s crop adaptation strategies identified a detailed list of climate change adaptation practices that are in congruence with the previous adaptation strategies described. These are (1) crop diversification, (2) change in cropping and planting pattern and calendar, (3) planting drought‐resilient crops, (4) mixed cropping, (5) improved irrigation efficiency, (6) adopting soil conservation measures, and (7) planning of trees and agroforestry [33].

**Figure 1:**
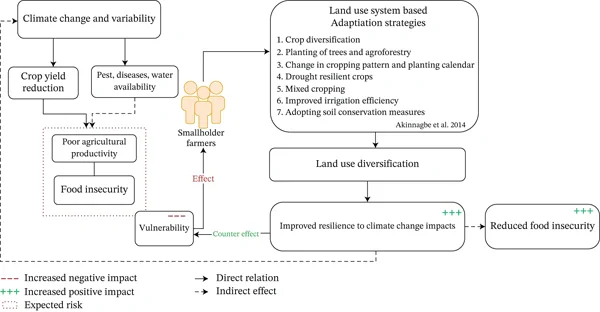
Conceptual framework of the study (*Source:* own illustration).

The conceptual framework of our study underlies the assumption that climate change and variability directly impact agricultural productivity posing high concern for food security while it also indirectly contributes to the same risk of food security by threatening water availability for agricultural production and increasing disease and pest outbreaks (Figure 1). This has a serious impact on the livelihood of smallholder farmers who rely on rain‐fed agriculture. At the same time, smallholder farmers practice diverse livelihood practices to adapt climate change impacts and increase resilience to climate change and climate variability‐induced risks that threaten food security. Furthermore, improved resilience to climate change impacts indirectly contributes to reducing food insecurity. With this background our study is aimed at analyzing the impact of land use diversification to improve the resilience of smallholder farmers to climate change and climate variability‐induced risks. For instance, diversification can directly reduce the risk of disease and pests associated with monoculture cultivation. The study assesses the contribution of land use diversification by analyzing the relative performance of three different land use systems and land diversification strategies in three regions of Ethiopia: Dinsho, Hawassa Zuria, and Fagita Lokoma districts.

## 3. Methodology

### 3.1. Study Area

This study assesses the relative performance of distinct land use systems in contributing to the resilience of smallholder farming communities against drought‐induced food insecurity in Ethiopia. The analytical framework proceeds in two sequential steps, moving from a broader zone‐level assessment to a focused district‐level investigation. In the first step, three zones were selected: Bale Zone (Oromia Region), Sidama Region (formerly part of the SNNPR Region), and Awi Zone (Amhara Region). These zones represent contrasting dominant land use systems monocrop/grassland–based agriculture in Bale, agroforestry in Sidama, and woodlot‐integrated systems in Awi. Zone‐level crop damage data [8] and food insecurity classifications (IPC [38]) were used to examine how these systems responded to the 2015–2016 El Niño–induced drought. This enables a comparative assessment of the relationship between dominant land use systems and drought‐related food insecurity outcomes.

In the second step, one representative district from each zone was selected for detailed analysis: Dinsho (Bale), Hawassa Zuria (Sidama), and Fagita Lokoma (Awi). These districts reflect the dominant land use systems identified at the zone level. Land use and land cover (LULC) classification based on Landsat 8 imagery (2015) was conducted to characterize land use composition during the drought period and to assess whether district‐level land use diversity aligns with food security patterns observed at the zone level.

The study adopts a purposive comparative case study design, selecting ecologically distinct land use systems across major agroecological zones. Although this enables structured comparison, the study areas differ in elevation, rainfall, market access, and population density. These differences are not controlled for but are considered intrinsic to the systems under study. Land use systems are therefore interpreted as embedded within broader agroecological and socioeconomic contexts and observed differences in food security outcomes reflect their combined effects.

The study does not isolate land use as the sole determinant of resilience. Instead, it examines whether patterns in crop damage, food aid priority classification, and Integrated Food Security Phase Classification (IPC) food insecurity phases are consistent with the hypothesis that diversified, tree‐integrated systems are associated with lower vulnerability to drought. Findings are context‐specific and should not be generalized without further analysis based on household‐level data. Table 1 summarizes the principal contextual variables that covary with land use type across the three cases. Comparisons in this study should therefore be read as land use‐system‐in‐context rather than as an isolated land use effect, and the discussion is framed accordingly.

**Table 1:** Contextual characteristics of the three case‐study districts and their zones.

District (zone, region)	Elevation (m a.s.l.)	Mean annual rainfall (mm)	Temperature (°C)	Dominant land use system	Main crops and products
Dinsho (Bale, Oromia)	2000–3600	1387	3.9–17.2	Monocropping/grazing	Wheat, barley, potato; livestock
Hawassa Zuria (Sidama)	1700–1850	1015	17–30	Homestead agroforestry	Enset, maize, coffee, khat, haricot bean
Fagita Lokoma (Awi, Amhara)	1800–2900	2395	24.0 (mean)	*Acacia decurrens* woodlot	Teff, barley, potato, pulses; charcoal

### 3.2. Case Study Districts

#### 3.2.1. Dinsho District (Bale Zone, Oromia Region)

Dinsho is located 7° 09 ^′^ 60.00 ^″^ N and 39° 54 ^′^ 59.99 ^″^ E with an elevation range from 2000 to 3600 m a.s.l. The annual average rainfall is 1387 mm, and the mean minimum and maximum temperature is 3.9°C and 17.2°C, respectively [19]. Mixed agriculture, annual crop production, and livestock rearing are the main livelihood practices in Dinsho and its surrounding districts [39, 40]. The agricultural landscape is known for its intensive wheat and barley production. Trees are found rarely in this agricultural landscape. In the district, land cover changes have been undergoing with the conversion of forest, bush, and grasslands to small‐scale annual croplands [41].

#### 3.2.2. Hawassa Zuria District (Sidama Region)

Hawassa Zuria is one of the districts in Sidama Regional State located at 6°55 ^′^58.6 ^″^ N and 38°29 ^′^48.8 ^″^ E. The area is located within an altitudinal range of 1700–1850 m a.s.l with an annual rainfall of 1015 mm. The minimum and maximum temperatures of the area is 17°C and 30°C, respectively [42]. Agroforestry is the major farming system of the area, and monocropping is practiced rarely. Particularly, the homestead agroforestry system (HAF) is the dominant farming system of the locality [20]. Maize (*Zea mays*), enset (*Ensete ventricosum*), khat (*Catha edulis*), coffee (*Coffea arabica*), and haricot bean (*Phaseolus vulgaris*) are the main food and cash crops practiced in the area [43]. Khat, coffee, and cattle are the leading sources of cash income, whereas maize and enset are the main food crops.

#### 3.2.3. Fagita Lokoma (Awi Zone in Amhara Region)

**Figure 2:**
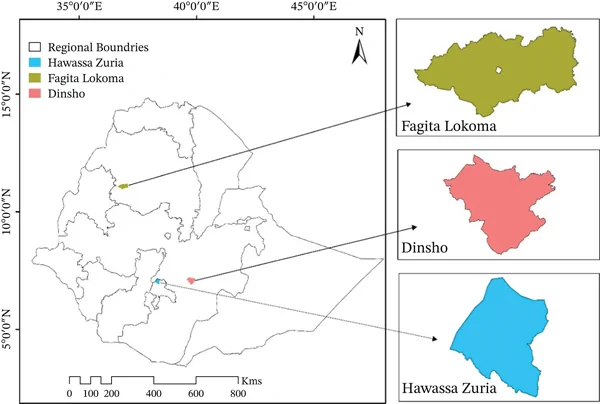
Location map of the case study sites (*Source:* own map).

Fagita Lokoma is located 10° 57 ^′^ ‐11° 11 ^′^ N and 36° 40 ^′^ ‐37° 05 ^′^ E with an elevation range from 1800 to 2900 m a.s.l [44]. The mean annual temperature is 24°C, and rainfall is 2395.3 mm. Land uses proximate to roads are converted to woodlot and homestead land uses are characterized by mixed plantation and crops, mixed farming system (crop and livestock production) is the main livelihood in the district and tef (*Eragrostis tef*), barely (*Hordum vulgare*), potato (*Solanum tuberosum*), bean (*P. vulgaris*), pea (*Pisum sativum*), and niger seed (*Guizotia abyssinica*) are the major crops cultivated in the district [34, 35, 45, 46]. Studies indicate that significant land use change is undergone in the district due to the adoption of *Acacia decurrens* plantations for biomass fuel [44–46]. The location of the three case study sites within Ethiopia is presented in Figure 2.

### 3.3. Data Source and Extraction Strategy

The 2015–2016 Ethiopian drought, associated with El Niño, was selected as a major climate shock to assess the resilience of different land use systems. Data were compiled from multiple sources to capture both agricultural impacts and food security outcomes. Crop damage and affected household data were obtained from the Ethiopian Statistical Service (ESS) (formerly CSA) for the 2015–2016 production year [47]. Food aid priority classifications were sourced from the Government of Ethiopia and the World Food Program (WFP) reports [48]. To complement these datasets, findings from the Inter‐Agency Humanitarian Evaluation (IAHE) of Ethiopia′s drought response (2015–2018), conducted in collaboration with the United Nations Office for the Coordination of Humanitarian Affairs (OCHA), were used for contextual validation. The IAHE applied a mixed‐methods approach, including key informant interviews, surveys, focus group discussions, quantitative analysis, and document review.

Food security conditions were further assessed using IPC data from 2019 (https://www.ipcinfo.org/). The use of IPC data from this period is partly driven by data availability, as nationally comparable IPC datasets for Ethiopia are only consistently available from 2019 onwards. In addition, the analysis is aimed at capturing lagged and persistent effects of the 2015–2016 drought, including recovery dynamics and structural vulnerability. This approach assumes that the impacts of severe drought may extend beyond the immediate shock period and continue to influence food security outcomes over time. Accordingly, IPC data are not interpreted as direct measures of the 2015–2016 drought impacts, but rather as indicative of postdrought system resilience and recovery trajectories.

At the district level, LULC classification was conducted using Landsat 8 imagery (2015) for Dinsho, Hawassa Zuria, and Fagita Lokoma. An exploratory field survey was used to validate land use patterns and assess the role of diversification in supporting household livelihoods. All data sources were triangulated to ensure consistency and contextual interpretation. Empirical and conceptual work on multiyear drought trajectories in subsistence farming systems documents persistent postshock effects through asset depletion, soil‐productivity decline, and disruption of livelihood strategies [49, 50], and the IAHE [51] evaluation confirms that the structural impacts of the 2015–2016 El Niño drought in Ethiopia persisted through 2018.

The field survey was conducted during 2019–2020 across all three case‐study districts and was reconnaissance‐level in scope. Participants were selected through purposive sampling. Fieldwork in each district comprised site visits, transect walks across representative land use mosaics, and semistructured key‐informant discussions on the dominant land uses of the area. Formal key‐informant discussions were held with three officials per district the head of the district office and the experts responsible for agricultural extension and for natural‐resource management and were supplemented by informal discussions with smallholder farmers practicing the dominant land use system, held opportunistically during transect walks and not recorded as structured interviews. The limited number of formal informants reflects the reconnaissance scope of the exercise. The qualitative observations produced were used (i) to assign labels to spectral clusters in the LULC classification; (ii) to contextualize the secondary CSA, IPC, and WFP datasets; and (iii) to identify locally salient livelihood strategies that are not captured by administrative data.

### 3.4. Data Analysis

Given the comparative case study design with three analytical units, the analysis is descriptive rather than inferential. Statistical hypothesis testing is not appropriate due to the limited number of cases and the use of administrative (population‐level) data [52]. Reported percentages represent aggregated values and are not subject to sampling error. The analysis therefore focuses on identifying consistent patterns across cases rather than establishing causal relationships. We acknowledge this as a study limitation and note that hypothesis testing would require household‐level survey data collected across a statistically adequate sample of farming systems.

Using data extracted from the [48, 51] report, we assessed the selected areas based on WFP′s priority classification for food aid needs. The classification ranges from 1 to 3, with Priority 1 indicating drought hotspot areas requiring food aid, whereas Priority 2 and 3 areas have progressively lower food aid needs. Areas classified as having no priority do not require food aid support. Additionally, we examined the food insecurity phase classification provided by the IPC. The IPC′s acute food insecurity scale assesses the severity of food insecurity at a specific point in time, particularly conditions that pose an immediate threat to lives or livelihoods, irrespective of underlying causes, context, or duration (Table 2). For Ethiopia, IPC data is available from 2019 onwards, offering detailed insights into food availability and access (https://www.ipcinfo.org/). From the data extracted from CSA, we analyzed crop damage/loss and households that reported being victims of crop damage in the area. The analysis considered crop damage occurring due to rainfall shortage to reference it to a functional effect of drought on crop damage and further to food insecurity leading to food aid priority areas.

**Table 2:** Food security phase classification criteria indicators of the Integrated Food Security Phase Classification Food security phase classification criteria indicators of the IPC (*Source:* https://www.ipcinfo.org/).

Phase	Description	Priority response
Phase 1: None/minimal	Households are able to meet essential food and nonfood needs without engaging in atypical and unsustainable strategies to access food and income.	Action required to build resilience and for disaster risk reduction
Phase 2: Stressed	Households have minimally adequate food consumption but are unable to afford some essential nonfood expenditures without engaging in stress‐coping strategies.	Action required for disaster risk reduction and to protect livelihoods
Phase 3: Crisis	Households either (i) have food consumption gaps that are reflected by high or above‐usual acute malnutrition or (ii) are marginally able to meet minimum food needs but only by depleting essential livelihood assets.	Protect livelihoods and reduce food consumption gaps
Phase 4: Emergency	Households either (i) have large food consumption gaps, which are reflected in very high acute malnutrition and excess mortality, or (ii) are able to mitigate these gaps but only by employing emergency livelihood strategies and liquidating assets.	Save lives and livelihoods
Phase 5: Catastrophe/famine	Households have an extreme lack of food and/or other basic needs even after full employment of coping strategies. Starvation, death, destitution, and extremely critical acute malnutrition levels are evident. (For famine classification, an area needs to have extreme critical levels of acute malnutrition and mortality).	Revert/prevent widespread death and total collapse of livelihoods

To assess drought impacts and food security outcomes, crop damage data, food aid priority classifications, and IPC food insecurity phases were analyzed comparatively across the three case study areas. A triangulation approach based on Denzin [53] was employed to examine whether consistent relationships emerge between land use systems, crop damage intensity, and food insecurity outcomes. This approach enhances the robustness of the analysis; however, findings are interpreted as context‐specific and indicative rather than statistically generalizable.

In place of statistical inference, the robustness of the comparative pattern was assessed in two ways. First, crop damage was expressed using three alternative metrics absolute area lost, area lost per 1000 zone inhabitants, and area lost per affected person and the ordering of the three zones was compared across them. Second, ordering was compared against two independently produced data streams: the WFP/IAHE food‐aid priority classification and the IPC 2019 phase distribution. A comparative claim is reported only where the ordering holds across metrics and across sources; where metrics diverge, no ranking between the zones concerned is claimed. Because the three zones differ in the share of their population engaged in cropping and in mean holding size, the area‐based measures are treated as the more directly comparable indicators of drought impact on production, and the population‐share measure is reported alongside them rather than in place of them. This procedure establishes robustness to metric and source choice only.

LULC classification was conducted using an unsupervised Weka K‐Means algorithm in Google Earth Engine, which grouped pixels into spectral clusters based on reflectance values. These clusters were subsequently reclassified into four land‐cover categories in QGIS. Class labels were assigned through expert visual interpretation, supported by two ancillary sources: (i) high‐resolution imagery in Google Earth for the same calendar period, used as the qualitative reference layer for label assignment, and (ii) the Dynamic World near‐real‐time land‐cover dataset, used only as an interpretive aid for ambiguous spectral clusters and not as an independent validation source. This combined approach integrates automated classification with contextual interpretation, as commonly applied in data‐scarce environments [54]. Due to the absence of ground‐truth data for the study period, a formal accuracy assessment (e.g., confusion matrix) was not conducted, and this limitation is acknowledged. This constraint is common in historical and data‐scarce land‐cover studies, where contemporaneous field reference data cannot be reconstructed after the fact; in such settings classification is commonly supported by expert visual interpretation of high‐resolution imagery and by local field knowledge rather than by a formal error matrix, and formal accuracy assessment of historical classifications is frequently infeasible where contemporaneous high‐resolution reference data are unavailable [54–56]. Kuma et al. [56] similarly relied on field observations and key‐informant interviews to assign land use classes in southern Ethiopia. Google Earth imagery has itself been shown to yield land‐cover classification accuracy comparable to commercial high‐resolution imagery Hu et al. [57] supporting its use here as the reference layer for expert visual validation of the classified land use classes. Following this practice, classification outputs were evaluated qualitatively through expert visual comparison with high‐resolution Google Earth imagery for the same calendar period, supplemented by field observations and key‐informant knowledge of the dominant land uses. The absence of a formal quantitative accuracy assessment is therefore acknowledged as a methodological limitation. To partially mitigate this limitation, classification outputs were qualitatively validated through expert visual comparison with high‐resolution imagery available in Google Earth for the same calendar period. Dynamic World was used only as an interpretive aid for ambiguous spectral clusters and not as an independent validation source. Classification outputs are therefore reported as indicative of dominant land‐cover patterns rather than as pixel‐level estimates suitable for quantitative change detection.

## 4. Results

### 4.1. Food Insecurity Phases and Crop Damages in the Study Sites

According to CSA [47], a total of 415,203 people across the three study areas experienced crop damage due to rainfall shortages during the 2015–2016 Meher season. The extent of impact varied markedly across land use systems. In Awi Zone (woodlot system), 190,203 people were affected, with a total crop loss of 3802 ha, corresponding to approximately 0.02 ha per affected person. In the Sidama Region (agroforestry system), 35,000 people were affected, and crop losses amounted to 6629 ha, equivalent to 0.19 ha per person. The most severe impact was observed in Bale Zone (monocrop/grassland system), where 190,000 people were affected, and 54,354 ha of cropland were lost, corresponding to 0.29 ha per person. To enable comparison across zones of unequal size, these figures are additionally normalized against projected 2016 zone populations (derived from the 2007 Ethiopian Census applying the CSA annual growth rate of 2.6%; Table 3). The three metrics capture different dimensions of exposure and do not order the zones identically. Bale records the highest damage intensity on both area‐based measures (30.76 ha per 1000 inhabitants; 0.29 ha per affected person). Awi records the largest share of population reporting damage (15.4%) but the smallest loss per affected person (0.02 ha), a combination indicating damage that was widely distributed but shallow in extent. Sidama records the lowest values on both population‐normalized measures (0.9% of the zone population affected; 1.78 ha per 1000 inhabitants). The population‐share metric should be read with caution: CSA affected‐person counts are compiled at holding level, and the three zones differ in the share of their population engaged in cropping and in mean holding size, so the area‐based measures are the more directly comparable indicators of drought impact on production.

**Table 3:** Crop damage from the 2015–2016 El Niño drought across three study zones, absolute and population‐normalized.

Zone	2016 projected population	People affected	% zone population affected	Ha damaged	Ha damaged per 1000 population	Ha per affected person
Awi	1,238,381	190,203	15.4%	3802	3.07	0.02
Sidama	3,721,832	35,000	0.9%	6629	1.78	0.19
Bale	1,766,960	190,000	10.8%	54,354	30.76	0.29

*Note: Source:* Crop damage and affected‐population figures from [47]. Population projections computed by the authors from CSA [58] census counts. Ha per affected person divides damage by the affected subpopulation rather than by total zone population. Reported for comparability with the absolute figures.

**Figure 3:**
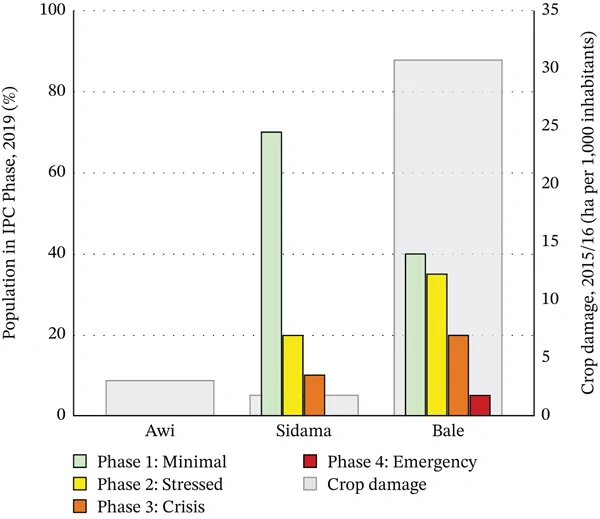
Crop damage due to rainfall shortage and food insecurity phases of affected population in the study sites. *Source:* [47] (crop damage data) and IPC 2019 (food insecurity phase classification).

Postdrought IPC phase classifications for 2019 further highlight spatial differences in food security outcomes. Awi Zone was not classified under any IPC food insecurity phase, as indicators of food access and availability did not meet the required thresholds. In Bale Zone, approximately 40% of the population (502,703 people) were classified in Phase 1 (Minimal), 35% (439,865) in Phase 2 (Stressed), 20% (251,351) in Phase 3 (Crisis), and 5% (62,838) in Phase 4 (Emergency). Overall, 60% of the population fell into Phase 2 or higher, with 25% in the more severe Phases 3 and 4. In Sidama, IPC data indicate that, out of an assessed population of 430,011, approximately 70% (301,008 people) were in Phase 1 (Minimal), 20% (86,002) in Phase 2 (Stressed), and 10% (43,001) in Phase 3 (Crisis). In total, 30% of the population was classified in Phase 2 or above. These distributions are presented alongside CSA‐reported crop damage in Figure 3, illustrating the relationship between drought‐induced crop losses and subsequent food security conditions across the study areas. Applying the robustness procedure described in the data analysis subsection, the position of Bale as the most severely affected zone holds across all three crop‐damage metrics and is consistent with both the WFP/IAHE priority classification and the IPC 2019 phase distribution. The relative positions of Awi and Sidama are metric‐dependent: Sidama records the lower area loss per 1000 inhabitants, whereas Awi records the lower loss per affected person and the higher share of population reporting damage. Based on this, there is no ranking between the two tree‐integrated systems.

### 4.2. Vulnerability of Smallholder Farmers to Climate‐Induced Risks

**Figure 4:**
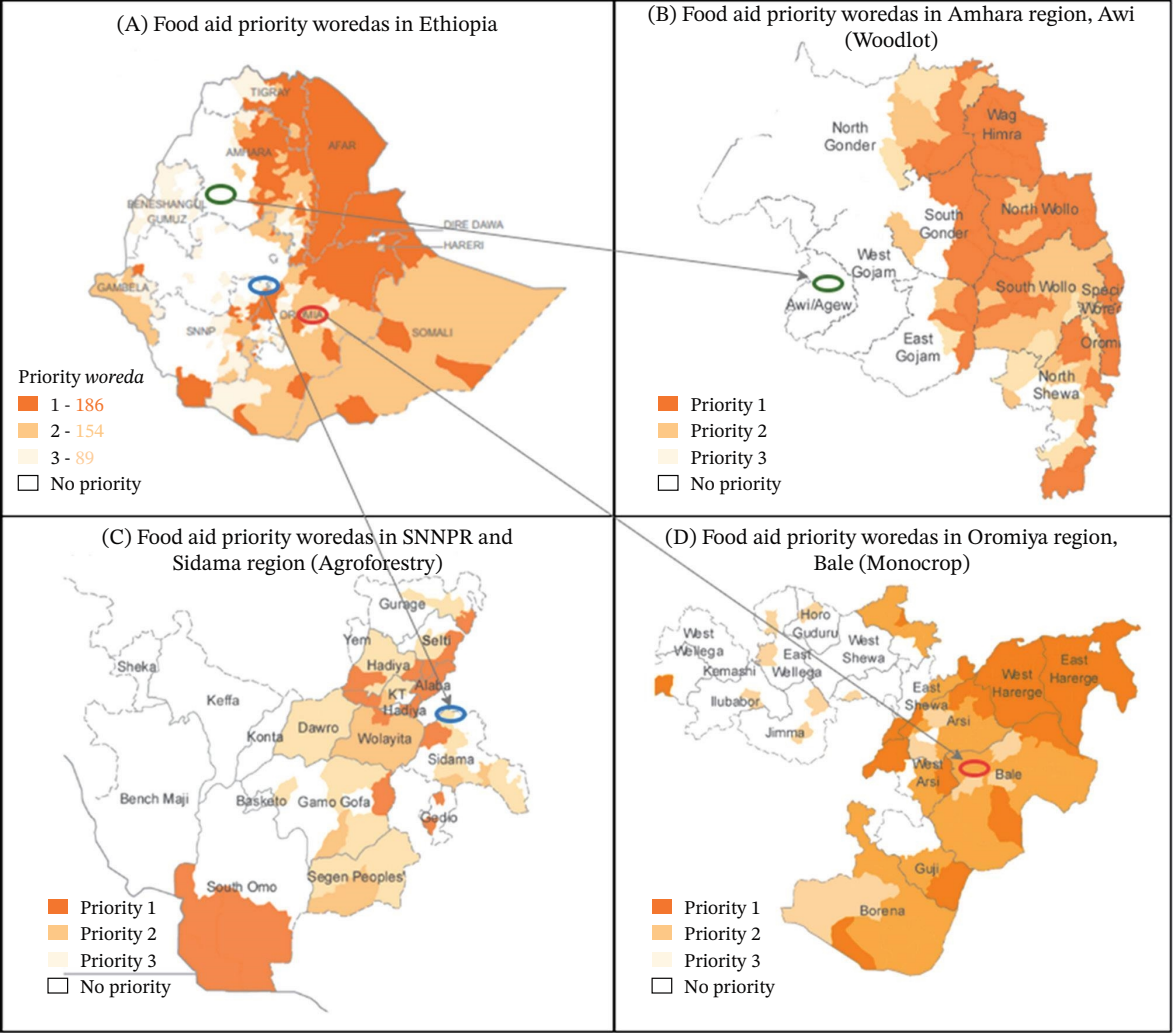
Spatial distribution of food aid priority and case study locations in Ethiopia. *Source:* [51]. (A) National distribution of food aid priority districts during the 2015–2016 drought (WFP classification: Priorities 1–3; no priority), with circles indicating selected case study districts. (B) Bale (monocrop/grassland) systems, (C) Sidama (agroforestry), and (D) Awi (woodlot).

Many regions of Ethiopia were affected by the drought in the 2015–2016 main cropping season. IAHE and OCHA [51] reported and produced a risk map showing the regions with high risk of food security in 2016. Figure 4 depicts the selected areas with the different land use practices with their respective priority class due to the drought occurrence and a threat in the food availability for the households. The figure is intended to support pattern‐based comparison rather than precise spatial quantification, emphasizing relative differences in food aid priority across systems. Variations in scale, labeling density, and administrative boundaries reflect the use of secondary data sources and do not affect the interpretation of overall patterns. The spatial distribution shows that the monocropping‐dominated district (Dinsho) is classified as Priority 2 for food aid, the agroforestry district (Hawassa Zuria) as Priority 3, and the woodlot‐dominated district (Fagita Lokoma) as no priority.

### 4.3. Land Use Practices of the Study Sites

**Figure 5:**
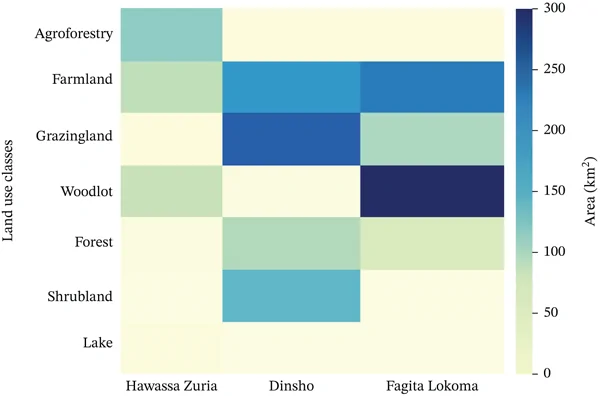
Land use and land cover variations in the selected case study sites during 2015.

The comparison of land use across Hawassa Zuria, Dinsho, and Fagita Lokoma reveals distinct patterns when expressed in percentages. In Hawassa Zuria, agroforestry dominates with 39.6% of the total land area, followed by farmland at 32.2% and woodlots at 27.2%, whereas the lake accounts for only 1%. In Dinsho, grazing land is the largest category at 34.5%, with farmland contributing 28.5%, shrubland 23%, and forest 14%, reflecting a strong pastoral and agricultural orientation. In Fagita Lokoma, woodlots are the most prominent, covering 45% of the landscape, followed by farmland at 30.3%, grazing land at 14.8%, and forest at 9.9%. Overall, farmland emerges as a consistently significant land use across all three sites, but each location shows a unique emphasis: Hawassa Zuria prioritizes agroforestry integration, Dinsho relies heavily on grazing systems, and Fagita Lokoma depends strongly on managed woodlots. Figure 5 presents the land use variations in the selected case study sites.

**Figure 6:**
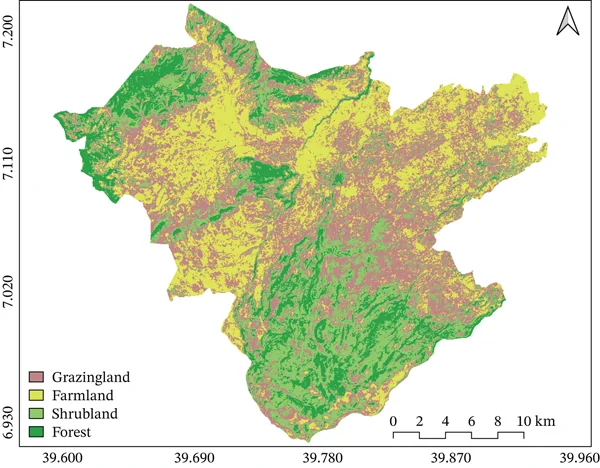
Land use land cover of Dinsho during the drought occurrence in 2015.

The land use distribution in Dinsho reveals a landscape predominantly shaped by grazing and agricultural systems. Grazing land constitutes the largest share, covering approximately 227.52 km^2^, followed by farmland at 188.05 km^2^. Shrubland, spanning 151.41 km^2^, reflects the significant presence of seminatural vegetation, whereas forested areas account for only 92.40 km^2^ (Figure 6). This configuration underscores the district′s strong dependence on pastoral and crop‐based livelihoods, which is consistent with field observations made during the exploratory survey.

**Figure 7:**
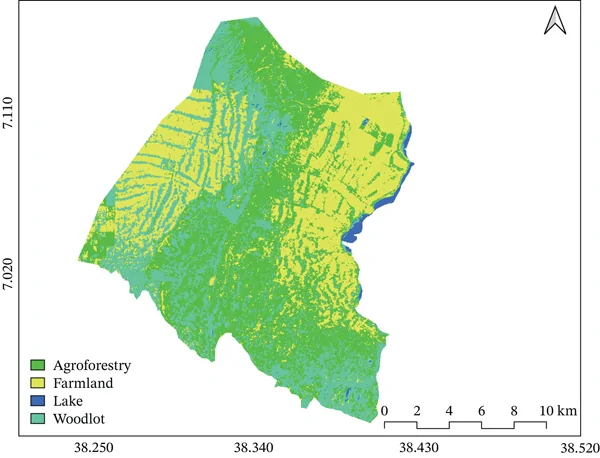
Land use land cover of Hawassa Zuria during the drought occurrence in 2015.

The land use profile of Hawassa Zuria reveals a landscape strongly shaped by agroforestry and agricultural production systems. Agroforestry constitutes the largest proportion of the area, covering approximately 117.93 km^2^, followed by farmland at 95.93 km^2^ (Figure 7). Woodlots make up an additional 80.99 km^2^, reflecting the widespread establishment of managed tree‐based systems, whereas the lake area remains minimal at only 3.06 km^2^. This composition underscores the centrality of tree‐integrated farming systems, with agroforestry serving as a dominant land management practice across the district. The substantial area under woodlots further indicates local investment in forest product provisioning and landscape restoration.

Hawassa Zuria′s land use system is highly diversified, and farm households are widely recognized for their homestead agroforestry practices. The agroforestry landscape is primarily dominated by cash crops such as coffee and khat, often intercropped with *Cordia africana* and staple crops like maize, forming the characteristic coffee‐based agroforestry system. Our exploratory survey indicates a gradual transition from the traditional enset‐based agroforestry toward intensified coffee and khat production, which informants attributed to the higher economic returns these cash crops offer. Despite this shift, enset remains a culturally and nutritionally significant crop, serving as a staple food and thus continuing to be cultivated around homesteads for household consumption.

**Figure 8:**
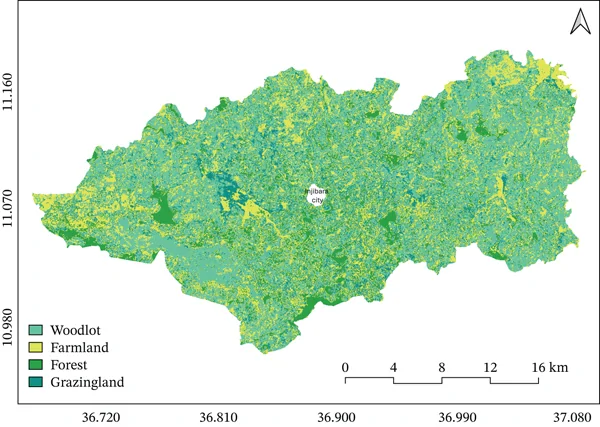
Land use land cover of Fagita Lokoma during the drought occurrence in 2015.

The land use profile of Fagita Lokoma reveals a landscape strongly influenced by both tree‐based systems and agricultural activities. Woodlots dominate the area, covering approximately 300.23 km^2^, followed by farmland at 202.22 km^2^. Grazing land constitutes 98.73 km^2^, whereas forested areas are relatively limited at 66.35 km^2^ (Figure 8). The substantial coverage of woodlots indicates an intensified reliance on managed tree cultivation, often integrated with crop production, reflecting a landscape shaped by both livelihood needs and evolving resource management strategies. Overall, the land use pattern of Fagita Lokoma illustrates a dynamic interaction between agricultural expansion, tree‐based resource utilization, and smallholder adaptation to economic opportunities.

Field observations show that *A. decurrens* woodlots have become the predominant land use system among smallholder farmers in the district. This shift is largely driven by the species′ rapid growth rate and high, immediate economic returns. During the establishment phase, farmers often intercrop *A. decurrens* with teff, the area′s major staple crop, particularly within the first 2 years. Once the trees become fully established, the woodlot occupies the land uninterrupted until reaching maturity, typically within 5–7 years. At the end of each rotation, farmers clear‐cut the plantation, and the harvested biomass is primarily used for charcoal production, which provides a significant and reliable source of household income.

## 5. Discussion

Because the classification could not be subjected to a formal quantitative accuracy assessment for the 2015 study period, these land‐cover patterns are interpreted as indicative of dominant land use configurations rather than as precise areal estimates. This interpretive, qualitatively validated approach is consistent with other land‐cover studies conducted in data‐scarce and historical contexts, where expert visual interpretation and local knowledge substitute for formal accuracy statistics when contemporaneous reference data are unavailable [55, 56]. The LULC analysis indicates that the three study areas have undergone substantial land use transitions, shaped by interacting ecological and socioeconomic drivers. In Bale, cropland expansion has occurred largely at the expense of forest and grassland ecosystems, reinforcing a monocropping production system [41, 59, 60]. Although such systems may achieve high productivity under favorable climatic conditions, they tend to reduce landscape heterogeneity and functional diversity. In contrast, Fagita Lokoma in Awi Zone has experienced rapid expansion of *A. decurrens* woodlots, primarily driven by the economic attractiveness of charcoal production [44–46]. Similarly, land use transitions in Sidama, particularly in Hawassa Zuria, reflect responses to population pressure, urban expansion, and market incentives, resulting in increasingly commercialized and diversified agroforestry systems [20, 44–46]. These findings are consistent with recent studies highlighting that land use systems in smallholder landscapes are dynamic and coevolve with livelihood strategies and market conditions [61, 62]. This variation underscores the diverse socioeconomic drivers shaping land use and highlights different opportunities that land diversification holds for building resilience to climate‐induced risks.

The results suggest that, within the study contexts, differences in food insecurity outcomes are associated with structural characteristics of land use systems. Monocropping systems in Bale were more affected during the 2015–2016 drought and were associated with a higher food aid priority. However, this pattern cannot be attributed to land use alone. Monocropping areas are often characterized by high dependence on seasonal rainfall, limited livelihood diversification, and greater exposure to input and output market fluctuations, all of which can amplify sensitivity to climatic shocks. This finding aligns with the WFP food aid hotspot priority ranking, which identifies monocrop‐producing areas as being at the highest risk, followed by agroforestry and woodlot‐dominated areas, respectively. In both agroforestry and woodlot‐dominated farming systems, annual and perennial crops are integrated, which moderates the extent of crop damage and reduces exposure to food insecurity risk [48, 51]. The HAF system in Sidama comprises diverse components including khat‐based, enset–cereal–vegetable, enset‐based, enset–coffee, and enset–livestock subsystems. Although the traditional food‐oriented enset‐based and enset–livestock systems have long prevailed, there is an observable shift toward more cash crop‐oriented systems like the khat‐based and combined food and cash crop‐oriented enset–cereal–vegetable systems, reflecting dynamic adaptation to evolving livelihood demands.

Diversification of the land uses was found to provide resilience to the farming household in times of shocks. The traditional enset based agroforestry is praised as climate smart practice which has the potential to endure long‐term drought season [63]. However, dependency of farmers on sole crop, Khat, systems than on the traditional diversified farming system is not a risk free transition in the face of climate change and dynamic market system [64, 65] Diversification, the intensification of current cropland through mixed‐cropping and intercropping, and cultivation of high value cash crops like khat are among the strategies adopted by farmers to cope with the reduced availability of cropland and the decreased productivity and food security performance of the newly evolved home garden systems [43] The khat and enset–cereal–vegetable systems were found better when compared with the traditional enset–coffee home garden systems [64, 65]. However, the home garden system change from traditional enset–coffee home garden systems to khat‐based and enset–cereal–vegetable systems is leading to a decline in livestock herds and a shift from manure to inorganic fertilizer use [64, 65].

Studies show that in areas where agriculture relies heavily on monoculture cropping systems, productivity is limited by various factors, including poor soil fertility, as well as the presence of crop pests and insects [66, 67]. However, crop diversification positively impacts soil health, primarily because it is linked to lower weed and insect pressures, reduced nitrogen fertilizer requirements (especially when leguminous crops are included), and decreased erosion due to the inclusion of cover crops [66, 68], which finally contributes to improved food security by contributing to improved productivity and farm income stability [69, 70]. In our case study site, *A. decurrens* woodlot plantation was recognized for its advantages of enhancing ecological sustainability. Ebabu et al. [71] uncovered that woodlot has lower runoff coefficient value (25%) as compared with grazing and cultivated lands (36% and 26%, respectively), implying that the woodlot practice reduces flood risks.

Although woodlot expansion in Awi Zone offers clear economic and ecological benefits, it also entails important trade‐offs. The conversion of cropland into *A*. *decurrens* plantations may reduce local food production, increasing reliance on market purchases and potentially exposing households to food price volatility [45, 46]. In addition, dependence on charcoal production introduces new vulnerabilities, as charcoal markets are often subject to regulatory restrictions, price fluctuations, and sustainability concerns. From an ecological perspective, woodlots can contribute positively to soil and water conservation. Studies show that *A. decurrens* plantations reduce runoff and erosion [71, 72]. However, intensification of woodlot production may also lead to longer‐term challenges, including nutrient depletion and reduced land availability for diversified cropping systems if not managed sustainably. These findings underscore that woodlot expansion should be understood as part of a broader land use strategy rather than a stand‐alone solution. Balancing woodlot production with food crops and agroforestry practices is essential to ensure that short‐term economic gains do not compromise long‐term food security and ecosystem sustainability. Similar trade‐offs between income diversification and food production have been reported in other smallholder contexts [61, 62].

The food aid priority classifications and IPC phase distributions observed across the three study districts are consistent with the hypothesis that land use diversification contributes to reduced district‐level food insecurity exposure during drought. The monocropping‐dominated landscape of Dinsho, characterized by crop‐ and grass‐based production systems, was classified at a higher food aid priority level and exhibited a greater proportion of its population in stress and crisis phases than the other two districts. Such systems are structurally more exposed to prolonged dry conditions because productivity is directly dependent on seasonal rainfall and the associated narrow range of livelihood sources. By contrast, the agroforestry system of Hawassa Zuria integrates drought‐tolerant perennial crops, including enset (from which staple foods kocho and bula are derived), with cash crops such as coffee and khat [64, 65]. The woodlot system of Fagita Lokoma, which provides diversified income streams from acacia‐derived wood and charcoal products, appears associated with lower food aid priority classification and absence of IPC phase classification. Tree‐integrated and multistrata land use systems may confer resilience through improved microclimatic regulation, soil moisture retention, and diversified livelihood sources that buffer against single‐season crop failure. Overall, the results suggest that land use diversification may enhance resilience through two key pathways: (i) stabilizing production through increased crop diversity and ecological functions, and (ii) improving income flexibility through market‐oriented outputs. However, the relative contribution of these pathways varies across systems and is shaped by local socioeconomic conditions.

## 6. Limitation

The comparative design is based on three purposively selected cases, which limits the scope for statistical inference. Hypothesis testing is not appropriate with only three population‐level administrative units, and the reported percentages represent aggregated values rather than sample estimates subject to sampling error. An additional limitation arises from the systematic differences among the three study areas in elevation, rainfall, market access, and population density. With only three observation units, these contextual factors cannot be statistically separated from land use type. Rainfall is particularly important, varying by more than twofold across the study areas, with the woodlot‐dominated district also being the wettest. Differences in baseline moisture availability may therefore contribute to the observed outcomes and cannot be ruled out. However, rainfall alone does not fully explain the observed ordering, as the driest district recorded a lower food‐aid priority than the wetter monocropping district. The comparisons should therefore be understood as contrasts among land use systems embedded within distinct environmental and socioeconomic contexts, rather than as estimates of an isolated land use effect.

The land‐cover classification also could not be subjected to a formal quantitative accuracy assessment because contemporaneous ground‐reference data for the 2015 classification period were unavailable and cannot be retrospectively reconstructed. The classification results are consequently interpreted as indicative of dominant land‐cover configurations rather than as pixel‐level estimates suitable for precise quantitative change detection. Nevertheless, the dominant patterns identified are broadly consistent with independently published assessments of the same districts, providing supporting evidence for their interpretation. Additionally, the field survey was designed as a reconnaissance‐level exercise to support triangulation and contextual validation rather than to provide a statistically representative household survey. Accordingly, the findings and associated inferences are framed at the district and zone levels. Establishing direct relationships between land use systems and household‐level resilience or food‐security outcomes would require systematically collected household‐level data, which were beyond the scope of this study.

The use of IPC data from 2019 to characterize postdrought food‐security conditions further relies on an assumed lag between the 2015–2016 drought‐related crop damage and subsequent food‐security outcomes. This assumption cannot be directly tested within the study because nationally comparable IPC data for Ethiopia are unavailable for the pre‐2019 period, and an earlier equivalent indicator could not be substituted. The corresponding interpretation should therefore be regarded as conditional on the plausibility of this lag effect rather than as evidence of a directly measured temporal relationship.

## 7. Conclusion

This study provides evidence that, within the selected case study areas, land use configuration is associated with differing levels of vulnerability to drought‐induced food insecurity. Monocropping systems, despite being productive under normal climatic conditions, were more affected during the 2015–2016 drought and were associated with higher food aid priority. In contrast, more diversified systems, particularly agroforestry and woodlot‐based land uses, were associated with lower crop damage and reduced food aid priority. These findings provide empirical support for the growing body of literature emphasizing the role of land use diversification in buffering climate risks and stabilizing livelihoods.

The findings suggest that, in the studied contexts, land use diversification may contribute to enhanced resilience by reducing exposure to climate‐related risks and providing alternative livelihood options. Furthermore, they highlight the potential role of diversified, tree‐based systems as part of context‐specific strategies for strengthening resilience to climate variability. The results of this study inform policymakers and stakeholders to strengthen and upscale the ongoing land use diversification practices to promote food security outcomes in the face of climate change. Strengthening policies that incentivize smallholder farmers to practice land use diversification can significantly contribute to achieving the intended goal of counteracting climate change‐induced risks and food insecurity. Furthermore, smallholder farmers and communities should be recognized for their contribution to global efforts of climate change mitigation and adaptation through land diversification, and more importantly, following tree‐based farming systems. Because the analysis is based on aggregated zone‐ and district‐level data, it cannot resolve causal relationships at the household level. Future research should therefore draw on household‐level panel data and longitudinal approaches to examine how land use practices and livelihood strategies interact over time to shape food security outcomes under conditions of climate variability.

## Author Contributions

We confirm that all listed authors made a substantial contribution to the research. Conceptualization: Shibire Bekele Eshetu; methodology: Shibire Bekele Eshetu and Haftom Hagos; formal analysis and investigation: Shibire Bekele Eshetu, Meselu Tegenie Mellaku, and Haftom Hagos; writing—original draft preparation: Shibire Bekele Eshetu, Meselu Tegenie Mellaku, and Katharina Löhr; writing—review and editing: Shibire Bekele Eshetu, Meselu Tegenie Mellaku, Haftom Hagos, Katharina Löhr, Negasi Solomon, and Stefan Sieber; funding acquisition: Shibire Bekele Eshetu and Stefan Sieber; resources: Shibire Bekele Eshetu, Katharina Löhr, and Stefan Sieber; supervision: Katharina Löhr and Stefan Sieber.

## Funding

This study was supported by the Alexander von Humboldt‐Stiftung (10.13039/100005156).

## Conflicts of Interest

The authors declare no conflicts of interest.

## Data Availability

The data that support the findings of this study are available from the corresponding author upon reasonable request.
